# URB447 Is Neuroprotective in Both Male and Female Rats after Neonatal Hypoxia–Ischemia and Enhances Neurogenesis in Females

**DOI:** 10.3390/ijms25031607

**Published:** 2024-01-28

**Authors:** Gorane Beldarrain, Marc Chillida, Enrique Hilario, Borja Herrero de la Parte, Antonia Álvarez, Daniel Alonso-Alconada

**Affiliations:** 1 Department of Cell Biology and Histology, School of Medicine and Nursing, University of the Basque Country (UPV/EHU), 48940 Leioa, Spain; 2 Department of Surgery and Radiology and Physical Medicine, Faculty of Medicine and Nursing, University of the Basque Country (UPV/EHU), 48940 Leioa, Spain

**Keywords:** neonatal hypoxia–ischemia, endocannabinoid system, neuroprotection, neurogenesis

## Abstract

The need for new and effective treatments for neonates suffering from hypoxia–ischemia is urgent, as the only implemented therapy in clinics is therapeutic hypothermia, only effective in 50% of cases. Cannabinoids may modulate neuronal development and brain plasticity, but further investigation is needed to better describe their implication as a neurorestorative therapy after neonatal HI. The cannabinoid URB447, a CB1 antagonist/CB2 agonist, has previously been shown to reduce brain injury after HI, but it is not clear whether sex may affect its neuroprotective and/or neurorestorative effect. Here, URB447 strongly reduced brain infarct, improved neuropathological score, and augmented proliferative capacity and neurogenic response in the damaged hemisphere. When analyzing these effects by sex, URB447 ameliorated brain damage in both males and females, and enhanced cell proliferation and the number of neuroblasts only in females, thus suggesting a neuroprotective effect in males and a double neuroprotective/neurorestorative effect in females.

## 1. Introduction

Perinatal hypoxia–ischemia (HI) often causes a brain condition known as hypoxic–ischemic encephalopathy (HIE), affecting around 2–3/1000 newborns in developed countries and 10–20/1000 newborns in low/middle-income countries [1,2,3]. Newborns who undergo HI may develop epilepsy, mental retardation, visual and hearing problems and cognitive or behavioral disorders, among other things, contributing significantly to overall disability worldwide [2]. As recent reports suggest that males are more vulnerable to suffering from prenatal anoxia, hemorrhage and infection [4,5,6], sex dimorphic responses to HI must be considered.

Currently, the only therapy available to treat HIE in hospitals is therapeutic hypothermia. Due to its limited efficacy, alternative neuroprotective strategies are being tested, although none have been approved for clinics yet. In recent years, the modulation of the endocannabinoid system (ECS) has been proposed as a potential therapy, as this system participates in processes like brain plasticity, learning, memory, neuronal development, energy balance and/or thermogenesis [7]. After harmful events like HI, the ECS also plays an important role in regulating glutamate excitotoxicity, oxidative stress and inflammation, mainly through the action of the cannabinoid receptors 1 and 2 (CB1 and CB2) [8,9,10].

CB1 receptors are mostly expressed in the central nervous system and are responsible for the regulation of excitotoxicity and oxidative stress after HI, while CB2 receptors appear mainly in immune system cells, regulating the inflammatory response caused by the injury [11]. Previous studies have focused on activating/inactivating these receptors to enhance the neuroprotective effect of the ECS, but have obtained controversial outcomes. CB1 receptor stimulation, for example, was neuroprotective in some in vitro and in vivo studies [12,13,14], but neurotoxic in others [15]. Moreover, CB1 antagonism can also have beneficial effects, as reported in a preclinical study using middle cerebral artery occlusion [16]. The modulation of the CB2 receptor, in contrast, has shown more consistent results, as its activation resulted in anti-inflammatory responses, whereas its antagonism has described no beneficial effects [17]. Further, the ECS may play a role in neurogenesis after HI [11], as its modulation may compensate cell death caused by the insult by promoting cell proliferation and tissue repair.

URB447 ({[4-amino-1-(4-chlorobenzyl)-2-methyl-5-phenyl-1H-pyrrole-3-yl](phenyl) methanone}) is a synthetic cannabinoid that binds to both CB1 and CB2 receptors, acting as simultaneous CB1-antagonist and CB2-agonist [18]. In neonatal rats, we previously showed that URB447 reduced brain injury and white matter demyelination [19], but little is known about its neuroprotective effect depending on sex after HI. In preclinical models of HI, sex differences are not fully clear: some authors have not observed any dimorphic effect on the injury [20], whereas others have found sex dimorphism in specific brain areas after HI, like the cortex [21,22]. Clinically, results seem more consistent, and male sex is considered a risk factor [23]. Further, the neuroprotective effect of therapies may differ depending on sex [24], just as may the neurogenic response of some treatments [25,26]. 

Generating new neural cells after HI may also help to ameliorate the effects of brain injury caused by HI, so discovering new therapies to enhance these processes is of great interest. Neurogenesis only remains in two areas after birth: the subventricular zone of the lateral ventricles and the subgranular zone of the dentate gyrus of the hippocampus [27,28,29]. As the neurogenic potential of the hippocampus may be affected after HI due to its particular vulnerability [30], its protection might help to maintain its neurogenic nature. 

In this work, we wanted (i) to explore the possible dimorphic effect of sex on HI and subsequent URB447 treatment and (ii) to evaluate the hippocampal neurogenic response after the administration of the cannabinoid in male and female neonatal rats.

## 2. Results

### 2.1. Body and Brain Weight

Data from postnatal day 7 (P7) body weights and P14 body and brain weights from each experimental group are shown in Table 1.

The day of the surgical procedure (P7), the weight of the animals was similar in all groups. Seven days after HI (P14), animals from the HI group had lower body weights when compared to sham (*p* < 0.0001 for both sexes; *p* < 0.05 for males and *p* < 0.01 for females). The HI+URB447 group showed lower body weight when compared to sham for all the pups (*p* < 0.01), but without differences when evaluated by sex or in comparison with HI animals. Brain weight reduction was evident in both sexes, males and females, from the HI group compared to sham (*p* < 0.0001), and so was the HI+URB447 group (*p* < 0.01 in both sexes and females; *p* < 0.001 in males). Differences between the HI and the HI+URB447 groups were observed when comparing both sexes together (*p* < 0.05) and in females (*p* < 0.01), with higher weights in the treated groups.

### 2.2. Brain Injury Assessment

#### 2.2.1. Hemispheric and Hippocampal Area Ratios

Hemispheric area ratios of both sexes together showed no signs of injury in sham animals, as the ratio values were close to 1 (1.01 ± 0.02). After HI, the areas of the ipsilateral hemispheres were reduced by a half (0.50 ± 0.26), being significantly lower than the sham ratios (*p* < 0.0001). The hemispheric ratios of the URB447-treated animals revealed similar values to the sham group (0.92 ± 0.14), being significantly higher than non-treated HI pups (*p* < 0.001).

**Figure 1:**
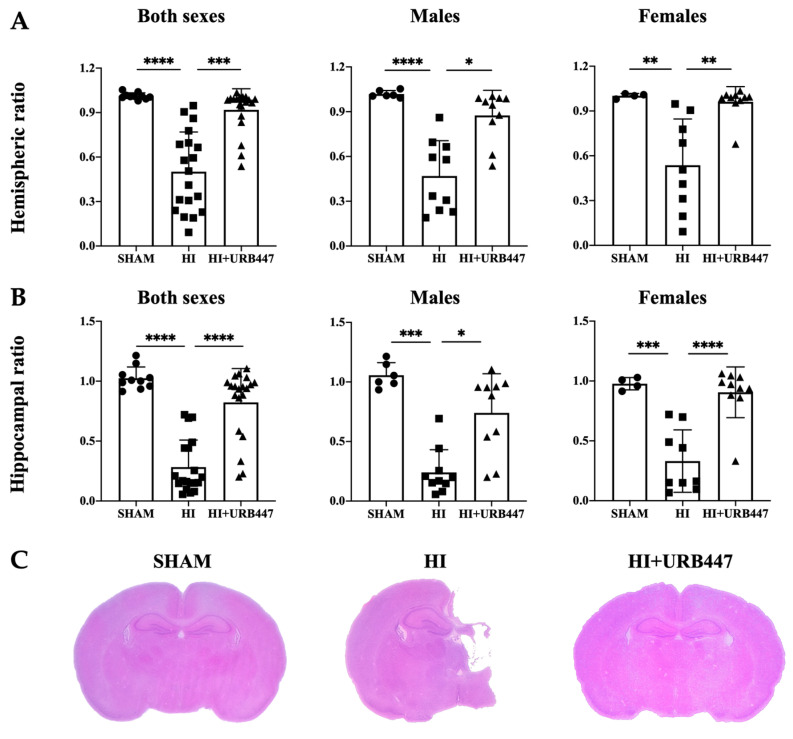
Effect of HI and URB447 treatment on (**A**) hemispheric and (**B**) hippocampal ratios from neonatal rats. Data analysis was first performed in both sexes together and then in males and females separately. HI reduced the hemispheric and hippocampal ratios, but subsequent URB447 treatment restored ratios to sham-like values in all cases. * *p* < 0.05; ** *p* < 0.01; *** *p* < 0.001; **** *p* < 0.0001 vs. HI. Representative low-magnification photographs (**C**) of brain sections from sham, HI and HI+URB447 treated animals. H&E staining.

When separating data by sex, the reduction in the ipsilateral hemisphere area was evident in both males (sham: 1.02 ± 0.02 vs. HI: 0.47 ± 0.24; *p* < 0.0001) and females (sham: 1.00 ± 0.02 vs. HI: 0.54 ± 0.1; *p* < 0.01) from the HI group. Again, the hemispheric ratios of URB447-treated animals (males: 0.87 ± 0.17; females: 0.96 ± 0.10) were closer to sham, and therefore significantly higher than HI-group ratios for both sexes (*p* < 0.05 for males and *p* < 0.01 for females). Results are shown in Figure 1A.

We further calculated the effect of the cannabinoid vs. HI depending on sex: the percentage of improvement for the hemispheric area ratios for males was 45.98%, and 43.75% for females. 

In the hippocampal area, sham animals also revealed ratios close to 1 (1.02 ± 0.09) when analyzing males and females together, a sign of no tissue loss. As previously observed for the ipsilateral hemisphere from non-treated HI pups, the hippocampal ratio was greatly reduced (0.28 ± 0.22), being significantly lower than sham (*p* < 0.0001). On the contrary, the hippocampal ratios of URB447-treated animals were significantly higher than the HI ones (0.82 ± 0.28; *p* < 0.0001), revealing fewer signs of injury and producing data similar to sham.

When analyzing the data of males and females separately, a similar protective pattern of URB447 was obtained. In the sham males or females, no signs of injury were observed (males: 1.06 ± 0.10; females: 0.98 ± 0.05), whereas HI hippocampal ratios were lowered by more than a half in both sexes (males: 0.24 ± 0.19, *p* < 0.001; females: 0.33 ± 0.26; *p* < 0.001). URB447 maintained hippocampal ratios to sham-like values in both males (0.74 ± 0.33) and females (0.90 ± 0.21), being significantly higher than those from HI in both sexes (*p* < 0.05 for males and *p* < 0.0001 for females). Results are shown in Figure 1B.

As we did with the hemispheric ratios, we also calculated a percentage of improvement for the hippocampal area ratios, being 67.57% for males and 63.33% for females. 

#### 2.2.2. Neuropathological Score

**Figure 2:**
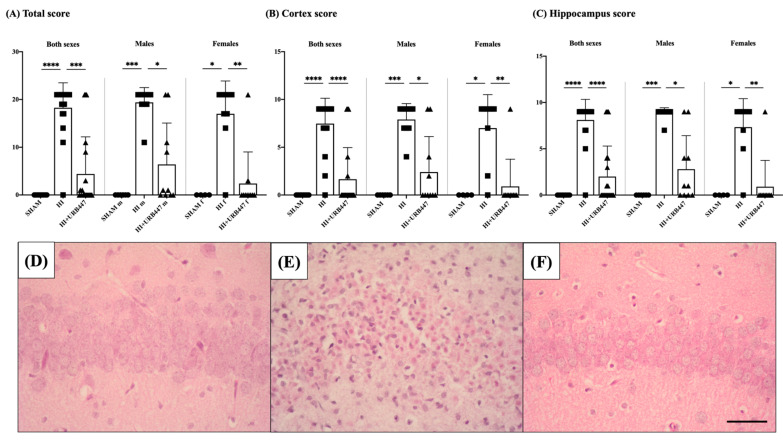
Effect of HI and URB447 on neuropathological score of total brain (**A**), cortex (**B**) and hippocampus (**C**). Data analysis was first performed in both sexes together and then in males and females separately. Non-treated HI animals showed the highest scores, whereas subsequent URB447 treatment restored scores to sham-like values in all cases. * *p* < 0.05; ** *p* < 0.01; *** *p* < 0.001; **** *p* < 0.0001 vs. HI. Representative microphotographs of the CA1 area of the hippocampus from sham (**D**), HI- (**E**) and HI+URB447-treated (**F**) animals. H&E staining. All images were taken at 40× magnification. Scale bar: 50 μm.

To further assess HI-induced brain injury and the potential neuroprotective effect of the cannabinoid URB447, we examined the samples using a semi-quantitative injury scoring system. In global/total neuropathological score, the highest damage was established when obtaining 21 points for the whole brain, whereas a regional assessment of cortical and hippocampal score may reach a maximum of 9 points. Results are shown in Figure 2.

In the total score for both sexes, the sham group received a mark of 0, as there was no visible tissue damage in these samples. In contrast, the HI group obtained a mark of 18.26 ± 2.60, significantly higher than sham (*p* < 0.0001). Animals treated with URB447 showed low signs of injury and received a score of 4.40 ± 7.78 points, significantly lower than the non-treated HI ones (*p* < 0.001) and similar to the values obtained for sham.

Similarly, when studying the total scores of male and female animals separately, HI-males (19.40 ± 3.10) and HI-females (17.00 ± 6.87) obtained total scores significantly higher than those from sham animals (*p* < 0.001 for males and *p* < 0.05 for females). Consistent with previous data, the beneficial effect of URB447 was observed in both sexes, as the scores were significantly lower in both males (6.40 ± 8.67; *p* < 0.05 vs. HI) and females (2.40 ± 6.60; *p* < 0.01 vs. HI) when compared to HI, and similar to sham. When evaluating the effect of the treatment compared to HI, we found that females reduced their scores more substantially (85.88%) than males (67.01%), although both sexes benefited from the treatment.

We then assessed the neuropathological score in the main affected brain areas after HI: the cortex and the hippocampus.

In the cortex, the HI group received a score of 7.47 ± 1.65 points, significantly higher than sham (*p* < 0.0001, which received 0 points), and also than the URB447-treated group (*p* < 0.0001, which received 1.65 ± 3.31 points). Sham and URB447-treated cortical scores were not different. When studying the two sexes separately, the cortical scores for HI-males (7.90 ± 166) and for HI-females (7.00 ± 3.50) were higher than sham (*p* < 0.001 for males and *p* < 0.05 for females). URB447-treated males and females, however, obtained significantly lower neuropathological scores vs. HI (males: 2.40 ± 3.72; *p* < 0.05; females: 0.90 ± 2.85; *p* < 0.01). As we found with the total score, the effect of the cannabinoid compared to HI in the cortex scores was 87.14% for females and 69.62% for males.

Lastly, the hippocampal neuropathological score of both sexes revealed that rat pups from the HI group had significantly higher neuropathological score values (8.10 ± 2.23; *p* < 0.0001) when compared to sham animals. Again, URB447-treated hippocampal scores were significantly lower than the HI ones (2.00 ± 3.29; *p* < 0.0001), with no difference to sham.

When evaluating males and females separately, the same pattern was observed: sham animals showed no injury signs in any sexes, whereas HI animals revealed high signs of damage, reaching nearly the maximum score in males (8.80 ± 0.63; *p* < 0.001) and females (7.33 ± 3.08; *p* < 0.05). URB447-treated rats, on the contrary, did not show signs of damage in the hippocampus, with significantly lower scores than the HI group in both males (2.80 ± 3.61; *p* < 0.05) and females (0.90 ± 2.85; *p* < 0.01), and no difference with sham pups. Results from the treated group and the HI group were compared, showing hippocampal score improvements in both females (87.72%) and males (68.18%).

### 2.3. Cell Proliferation

We next used the nuclear marker Ki67 to evaluate cell proliferation.

**Figure 3:**
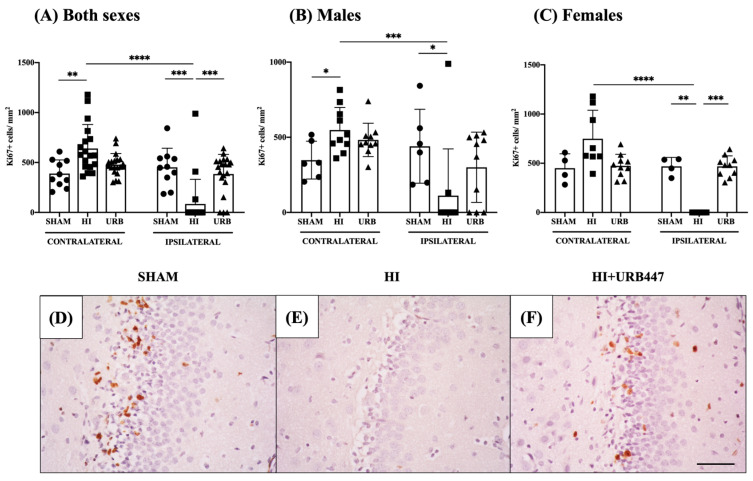
Effect of HI and URB447 on cell proliferation revealed by Ki67+ cell counts. Data analysis was first performed in both sexes together and then in males and females separately. Graphs show Ki67+ cells per mm^2^ in both sexes (**A**), males (**B**) and females (**C**). * *p* < 0.05; ** *p* < 0.01; *** *p* < 0.001; **** *p* < 0.0001 vs. HI. Representative microphotographs showing Ki67 expression in the subgranular zone (SGZ) of the ipsilateral hippocampi from sham (**D**), HI (**E**) and HI+URB447-treated (**F**) animals. Ki67+ cells appear in brown. All images were taken at 40× magnification. Scale bar: 50 μm.

First, we analyzed the differences in the number of proliferating Ki67+ cells between the contralateral and the ipsilateral dentate gyrus of each group (i.e., Sham-C vs. Sham-I; HI-C vs. HI-I; HI+URB447-C vs. HI+URB447-I) for both sexes together (Figure 3A).

In sham animals, there were no differences in cell counts between the contralateral (389 ± 136 cells/mm^2^) and ipsilateral (452 ± 191 cells/mm^2^) sides. The HI group, in contrast, showed reduced Ki67+ cells on the ipsilateral hippocampi (85 ± 246 cells/mm^2^), when compared to the contralateral side (641 ± 239 cells/mm^2^; *p* < 0.0001). In URB447-treated animals, there were no differences between the contralateral (478 ± 111 cells/mm^2^) and ipsilateral (384 ± 197 cells/mm^2^) hippocampi. 

We then compared the results of the different experimental groups to assess the effect of HI and the treatment of URB447 in cell proliferation from contralateral (i.e., Sham-C vs. HI-C vs. HI+URB447-C) and ipsilateral (Sham-I vs. HI-I vs. HI+URB447-I) hippocampi for both sexes together.

In the contralateral hippocampi, HI animals had higher Ki67+ cells (641 ± 239 cells/mm^2^) compared to sham (389 ± 136 cells/mm^2^; *p* < 0.01). The number of proliferating cells was similar in the contralateral dentate gyri of HI and HI+URB447 groups.

When evaluating the ipsilateral hippocampi, HI reduced the Ki67+ cells (85 ± 246 cells/mm^2^; *p* < 0.001 vs. Sham). This decrease in cell proliferation induced by HI in the ipsilateral hippocampi was restored after URB447 treatment, showing higher Ki67+ counts in HI+URB447 animals (384 ± 197 cells/mm^2^; *p* < 0.001 vs. HI) and similarity to sham. 

Next, we compared the effect of sex in cell proliferation in the three experimental groups.

In males (Figure 3B), the number of proliferating cells in the sham group was similar in both hemispheres (contralateral: 348 ± 125; ipsilateral: 440 ± 246). In the HI group, ipsilateral cell counts (HI-I: 112 ± 311) revealed a statistically significant reduction in proliferating cells compared to contralateral (HI-C: 548 ± 150; *p* < 0.001). URB447-treated pups revealed no significant differences in contralateral (482 ± 111) and ipsilateral (300 ± 234) Ki67+ cells. 

We also compared the effect of HI and URB447 in cell proliferation from contralateral (i.e., Sham-C vs. HI-C vs. HI+URB447-C) and ipsilateral (Sham-I vs. HI-I vs. HI+URB447-I) hippocampi for males. The number of proliferating cells was significantly higher in HI animals compared to sham (Sham-C: 348 ± 126 vs. HI-C: 548 ± 150 cells/mm^2^; *p* < 0.05). The number of proliferating cells was similar in the contralateral dentate gyri of HI and HI+URB447. In the male ipsilateral hippocampi, on the contrary, we found reduced Ki67+ cells in the HI group compared to sham (Sham-I: 440 ± 246 vs. HI-I: 112 ± 311 cells/mm^2^; *p* < 0.05). URB447 treatment was not able to increase the number of proliferating cells in the ipsilateral hippocampi (300 ± 234 cells/mm^2^) when compared to HI.

In females (Figure 3C), the number of proliferating cells in the sham group was similar in both hemispheres (contralateral: 450 ± 146; ipsilateral: 469 ± 91). In the HI group, ipsilateral cell counts reduced significantly (*p* < 0.0001) compared to contralateral. Again, URB447-treated pups revealed no significant differences in contralateral (473 ± 117) and ipsilateral (469 ± 106) Ki67+ cells.

We also compared the effect of HI and URB447 in cell proliferation from contralateral (i.e., Sham-C vs. HI-C vs. HI+URB447-C) and ipsilateral (Sham-I vs. HI-I vs. HI+URB447-I) hippocampi for females. HI significantly reduced cell proliferation compared to sham (Sham-I: 469 ± 91 vs. HI-I: 0 ± 0 cells/mm^2^; *p* < 0.01), as it did in males. However, this reduction in cell proliferation was reverted in URB447-treated female rats, with Ki67+ cell counts (HI+URB447: 469 ± 106) significantly higher than non-treated HI pups (*p* < 0.001), and similar to sham. 

### 2.4. Quantification of Neuroblasts

**Figure 4:**
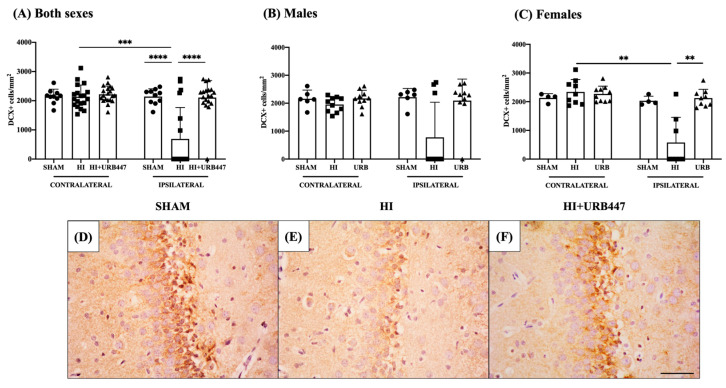
Effect of HI and URB447 on neurogenesis revealed by DCX+ cell counts. Data analysis was first performed in both sexes together and then in males and females separately. Graphs show DCX+ cells per mm^2^ in both sexes (**A**), males (**B**) and females (**C**). ** *p* < 0.01; *** *p* < 0.001; **** *p* < 0.0001 vs. HI. Images show representative photographs of the DCX expression in the SGZ of the ipsilateral hippocampi from sham (**D**), HI- (**E**) and HI+URB447-treated (**F**) animals. DCX+ cells appear marked in dark brown. All images were taken at 40× magnification. Scale bar: 50 μm.

We further wanted to explore the possible effect of HI and URB447 on neurogenesis, so we analyzed doublecortin (DCX) as a specific marker of neuroblasts in the neurogenic niche of the hippocampal subgranular zone (SGZ). Results are shown in Figure 4.

First, we counted the number of DCX+ neuroblasts in the contralateral and ipsilateral hippocampi of each group (i.e., Sham-C vs. Sham-I; HI-C vs. HI-I; HI+URB447-C vs. HI+URB447-I) for both sexes together (Figure 4A). In sham animals, there were no differences between the contralateral (2148 ± 247 cells/mm^2^) and ipsilateral (2142 ± 267 cells/mm^2^) sides. The HI group, in contrast, showed reduced DCX+ cells on the ipsilateral hippocampi (690 ± 1077 cells/mm^2^), significantly lower than the HI contralateral (2137 ± 398 cells/mm^2^; *p* < 0.001). In URB447-treated animals, there were no differences in DCX+ cell counts between the contralateral (2220 ± 280 cells/mm^2^) and ipsilateral (2108 ± 583 cells/mm^2^) hippocampi.

We then compared the results of the different experimental groups to assess the effect of HI and the treatment of URB447 in DCX cell counts from contralateral (i.e., Sham-C vs. HI-C vs. HI+URB447-C) and ipsilateral (Sham-I vs. HI-I vs. HI+URB447-I) hippocampi for both sexes together. In the contralateral hippocampi, we found no significant differences between any of the groups. When evaluating the ipsilateral hippocampi, DCX+ cells were only reduced in HI animals (Sham: 2142 ± 267 vs. HI: 690 ± 1077 cells/mm^2^; *p* < 0.0001). This reduction in neuroblasts was restored when treating animals with URB447 (HI+URB447: 2108 ± 583 cells/mm^2^), showing significantly higher DCX+ cells compared to HI (*p* < 0.0001), and similar to sham.

Next, we compared the effects of HI and the cannabinoid treatment, separating animals by sex.

In males (Figure 4B), the number of neuroblasts in the sham group was similar in both hemispheres (contralateral: 2160 ± 310; ipsilateral: 2215 ± 311). In the HI group, ipsilateral cell counts (HI-C: 1953 ± 267) revealed a not statistically significant reduction in neuroblasts compared to contralateral (HI-I: 778 ± 1256 cells/mm^2^). URB447-treated pups revealed no significant differences in contralateral (2169 ± 293 cells/mm^2^) and ipsilateral (2092 ± 773 cells/mm^2^) DCX+ cells. URB447-treated male rats did not show higher numbers of neuroblasts in the ipsilateral SGZ compared with HI rats.

In females (Figure 4C), all groups showed similar values for DCX+ cell counts except the HI ipsilateral hippocampi, whose counts (580 ± 873 cells/mm^2^) were significantly lower (*p* < 0.01) than in the contralateral ones (2341 ± 434 cells/mm^2^). Such a reduction was reverted by URB447 treatment, showing higher DCX cell counts (HI+URB447-I: 2126 ± 304 cells/mm^2^) than HI animals (*p* < 0.01), and similar ones to sham.

## 3. Discussion

In the present work, the administration of the cannabinoid URB447 after HI in neonatal rats revealed strong neuroprotective and neurorestorative effects, as it reduced brain infarct and global and regional neuropathological damage, together with increased cell proliferation and neuroblast number in the dentate gyrus. We also assessed whether sex would influence the therapeutic effect of the treatment: our results suggest that the cannabinoid was neuroprotective for both sexes, with stronger neurogenic benefit in females. 

In the last few years, several authors have noted that HI might affect males and females differently [6,31,32,33,34], which prompted us to separate our data by sex to evaluate a possible dimorphic response in brain damage after HI and subsequent URB447 treatment. Clinically, male sex is nowadays considered a risk factor in perinatal brain injury [23]. Here, non-treated HI males and females revealed similar post mortem neuropathology, despite demonstrating a trend towards more histological damage in males. In preclinical models, other authors have reported similar results to ours, with no sex differences in lesion size or tissue atrophy, where histological brain damage associated with HI was comparable across sex [20,35,36,37]. The pathophysiology of neonatal HI is complex and not fully understood yet, and so are the mechanisms leading to potential sexual differences; further investigation is needed to understand the basis of sexual dimorphism after HI.

The need for novel and effective therapies to treat neonatal hypoxic–ischemic encephalopathy is urgent. However, research works do not usually take into consideration sex dimorphism in their experimental designing, despite sex possibly being a crucial factor when evaluating the therapeutic effectiveness of neuroprotectants. We previously reported that the cannabinoid URB447 helped mitigate brain injury and white matter demyelination following neonatal HI [19], so we further wanted to characterize sex-specific outcomes in response to URB447 treatment. The neuroprotection parameters (hemispheric and hippocampal ratios and global and regional neuropathological scores) revealed increased benefit in females, but both treated males and females recovered the histological values observed for sham. Very few studies have evaluated the therapeutic effect of cannabinoids based on sex after acute brain damage. In a traumatic brain injury model, ∆9-tetrahydrocannabinol treatment impaired motor function and increased levels of cytokine interleukin-6 in males only [38], thus suggesting sex-specific cannabinoid effects. Together with the hormonal modulation of the ECS [39], different responses to cannabinoid administration on males and females have been described [40], and also sex differences in CB1 and CB2 receptor expression: female rats showed higher levels of CB1 and CB2 mRNA in the amygdala, hypothalamus, hippocampus and cortex compared to males under control conditions [41], the last two being regions affected by HI in our model and evaluated here.

Most of the studies assessing the therapeutic effect of the ECS after neonatal brain injury have focused on neuroprotection [19,42,43], but little is known about the potential of cannabinoids as brain-tissue-repairing therapies after HI. Moreover, the neurogenic response of the neonatal brain after damage is still not fully clear, with works suggesting that HI can enhance neurogenesis [44,45], while others report reduced cell proliferation [46,47].

Here, we wanted to explore the potential of URB447 as a neurogenic therapy, and showed both enhanced cell proliferation and an increased number of neuroblasts, thus suggesting a double neuroprotective/neurorestorative effect of the cannabinoid after HI. Different interactions with CB1 and CB2 receptors have previously been reported to modulate neurogenesis, including the increased proliferation of neural progenitors [48] and migration [49]. In the context of neonatal HI, we and others have described increased cell proliferation in the hippocampal dentate gyrus [46] and subventricular zone [50] by CB1 agonists 2-AG and WIN55,212-2, respectively. However, CB1 agonism may not be neuroprotective in certain types of neurons (like GABAergic) [51], so caution must be taken when choosing the cannabinoid to test. In this work, we opted for a CB1 antagonist to discard possible non-desirable neurotoxic or side effects. Further, CB2 receptor activation is also related to the enhancement of neurogenesis [48,49], as CB2-deficient mice showed impaired neural progenitor proliferation [48]. URB447 is also a CB2 agonist, so this cannabinoid may be a promising therapy targeting both neuroprotection and neuroregeneration after HI with a single molecule. In line with our results, Avraham and collaborators observed that the inhibition of neurogenesis was reverted in rodents after using a CB2 agonist [52]. CB2 expression levels are higher in less-differentiated cells [53,54], and CB2 receptor inhibition impaired neuroblast migration after stroke in mice [55].

We also wanted to test whether sex could affect neurogenesis after injury and subsequent URB447 administration. In non-pathological situations, some authors have reported that males generate more new cells in the hippocampus in the first postnatal week [56,57], whereas others have noted higher proliferation levels in females [58,59]. Here, we first showed that HI generated a similar reduction in cell proliferation and neuroblast counts for both sexes. When evaluating the neurogenic effect of the cannabinoid, females showed increased cell proliferation and neuroblast counts when comparing to non-treated pups, a response not observed for males. During brain development, sex may account for brain dimorphism [60,61,62], thus becoming a potential factor influencing different responses to damage and ulterior neurorestorative outcomes to cannabinoids or to other treatments.

In our work, we observed augmented Ki67+ proliferating cells in the contralateral hemisphere of non-treated HI animals, a possible compensatory mechanism of the non-damaged hemisphere in response to injury, as previously reported [63]. In our model, animals are exposed to systemic hypoxia, which also affects the contralateral hemisphere. At low oxygen concentrations, hypoxia-inducible factor-1α (HIF-1α) may promote neural stem cell proliferation [64], which may account for the observed increase in Ki67+ cells. 

Based on the results presented, some study limitations should be considered. The present work revealed important differences in the responses of males and females to HI and URB447 treatment, but the pathways involved could not be reported by the histological methods used. To better understand the mechanisms that follow HI and the treatment with the cannabinoid, further experiments should be performed in future works, which may include the analysis of HIF-1α expression. Also, long-term studies evaluating the effect of URB447 on brain neuroprotection/neuro-regeneration after HI must be considered, as similarly reported for 2-AG [46].

In conclusion, URB447 showed robust neuroprotective effects in male and female rats subjected to HI, as both sexes showed reduced brain infarct areas and improved total and regional neuropathological scores. Not only that, URB447 also enhanced neurogenesis in female rats, based on increased proliferation and neuroblast cell counts after treatment with the cannabinoid. Thus, our results support the role of URB447 as a double neuroprotective and neuroregenerative treatment, although further investigation is needed to fully understand the mechanisms behind these effects.

## 4. Materials and Methods

All experiments were in accordance with the European Union regulations for animal research (Directive 86/609/EEC) and approved by the Animal Welfare Committee of the University of the Basque Country.

### 4.1. Hypoxia–Ischemia

On postnatal day 1 (P1), Sprague Dawley rats were normalized to 10 pups per litter. On P7, rat pups were randomized into the three experimental groups included in this work. HI was performed in neonatal rats as described before [35,65,66,67,68] with minor modifications. Briefly, rat pups were weighed and anesthetized with isoflurane (4% for induction, 1% for maintenance) and the left common carotid artery was ligated in two locations using 6-0 surgical silk and then cauterized to ensure no blood circulation. After the surgical procedure, which did not exceed ten minutes per animal, pups were allowed to recover for 1 h by returning to their dams. Once the recovery interval finished, the induction of hypoxia was performed, placing the pups in humidified containers perfused with an 8% oxygen/92% nitrogen gas mixture for 2 h and maintained at 36 °C. 

Once the hypoxia period was finished, rats belonging to the experimental group treated with the cannabinoid URB447 (HI+URB447; *n* = 20, 10 males and 10 females) received a single i.p. dose of 1 mg/kg of URB447 3 h after the end of the hypoxia. Non-treated hypoxic–ischemic animals (HI; *n* = 19, 10 males and 9 females) received the same volume of vehicle as the treated group. Sham animals (*n* = 10, 6 males and 4 females) underwent the same surgical procedure but without artery ligation or hypoxia. Rat pups were returned to their dams until P14, when euthanized per protocol. 

### 4.2. Drug Preparation

URB447 was purchased from Cayman Chemical (Ann Arbor, MI, USA), dissolved in 1:9 PBS:DMSO and injected to a final concentration of 1 mg/kg.

### 4.3. Brain Processing and Tissue Preparation

At P14, animals were sacrificed via a lethal injection of sodium pentobarbital and perfusion-fixed with phosphate-buffered saline (PBS) followed by 4% paraformaldehyde in 0.1 M-PBS. Brains were removed and immersed again in 4% paraformaldehyde in 0.1 M-PBS solution at 4 °C overnight. After post-fixation, brain tissue blocks were prepared, dehydrated and embedded in paraffin.

For histological analysis, 5 μm sections were cut at the level of the mid-dorsal hippocampus and thalamus (Bregma −1.80 mm) according to Khazipov et al., 2015 [66]. Some of these sections were stained with Hematoxylin and Eosin (H&E) for brain injury assessment and other sections were used for Ki67 and DCX immunohistochemistry. 

### 4.4. Brain Injury Assessment

To assess HI-induced brain injury and the possible effect of the cannabinoid, we evaluated the ratios of non-infarcted hemispheric and hippocampal tissue areas and performed a neuropathological score for the whole brain, cortex and hippocampus for both contralateral (non-damaged) and ipsilateral (damaged) hemispheres.

#### 4.4.1. Hemispheric and Hippocampal Ratios

H&E-stained full brain slices were scanned, and the area of each hemisphere was measured manually using Fiji/ImageJ image software version 1.53k (National Institutes of Health, Bethesda, MD, USA). The hemispheric ratio was calculated in each slide by dividing the ipsilateral (left, damaged) and the contralateral (right, non-damaged) hemisphere areas. For hippocampal ratios, the same procedure was followed using the areas of the ipsilateral and contralateral hippocampi. To measure the effect of the cannabinoid URB447 compared to HI, a percentage of improvement was calculated for both hemispheric and hippocampal area ratios of males and females. The percentage was obtained following this formula: [(Ratio URB447 − Ratio HI)/Ratio URB447] × 100.

#### 4.4.2. Neuropathological Scores

In each slice, a semiquantitative scoring system (modified from [35,67]) was used to evaluate the extent of brain damage both globally and regionally in the parietal cortex, CA1, CA2/3 and dentate gyrus areas of the hippocampus. Brain injury was graded as follows: macroscopic damage (0–3); parietal cortex affectation (0 = no observable injury; 2 = a few small isolated groups of injured cells; 4 = several larger groups of injured cells, mild infarction; 7 = moderate confluent infarction; 9 = extensive confluent infarction); hippocampal damage in each CA-1, CA2/3 and dentate gyrus regions (0 = no observable injury; 1 = mild infarct; 2 = moderate infarct, 3 = observable cell infarction). The total score was calculated by adding the macroscopic brain injury score and the region-specific injury score, thus with 21 being the maximum score (most damaged) and 0 the minimum (non-damaged). To measure the effect of the cannabinoid compared to HI, a percentage of improvement was calculated for total and regional scores of males and females. The percentage was obtained following the next formula: [(Score HI − Score URB447)/Score HI] × 100.

### 4.5. Immunohistochemistry and Cell Counting

Briefly, 5 μm sections of brain samples were used for the immunostaining of Ki67 (cell proliferation marker) and DCX (Doublecortin, neuroblast marker). Samples were deparaffinized, rehydrated and immersed in a sodium citrate solution (10 mM sodium citrate + 0.05% Tween20 in distilled water at pH 6) that was boiled three times and then kept at 95–98 °C for 20 min for antigen retrieval. Once cooled at room temperature, the endogenous peroxidase was blocked by treating the samples with 3% H_2_O_2_ in PBS. After washing with PBS, slices were incubated in a blocking solution (5% bovine serum albumin + 0.4% Triton X-100 in PBS) for 1 h. Once the incubation period was finished, samples were washed again with PBS and incubated at 4 °C overnight with the corresponding primary antibody: Ki67 (1:50, BD Pharmingen; #550609) or DCX (1:50; Santa Cruz Biotechnology; sc-271390). The next day, slices were washed 3 times with PBS and incubated with the secondary antibody biotin conjugated (goat anti-mouse IgG H + L, #31800, Invitrogen, Thermo Fisher, Waltham, MA, USA) for 30 min. After incubation, samples were revealed using diaminobenzidine, counterstained with hematoxylin and mounted with DPX. Negative controls received identical treatment except for the omission of primary antibodies and showed no specific staining.

The quantification of Ki67- and DCX-positive cells was performed in the hippocampal SGZ of each hemisphere on 5 non-overlapping microphotographs at 40× magnification (10 photographs per sample) using an Olympus BX-50 light microscope. In each photograph, the area of the SGZ was measured and the number of positive cells was manually counted using Fiji/ImageJ image software version 1.53k (National Institutes of Health, Bethesda, MD, USA). Values are given as Ki67- or DCX-positive cells per mm^2^.

### 4.6. Statistical Analysis

The normality of the figures was analyzed using the D’Agostino–Pearson test. For parametric data, a two-tailed, unpaired Student’s *t*-test (to compare contralateral vs. ipsilateral hemispheres and males vs. females) and one-way analysis of variance (ANOVA) with Tukey’s multiple comparisons test (to compare the three experimental groups) were performed. In the same way, non-parametric data were analyzed using Mann–Whitney or Kruskal–Wallis tests with Dunn’s multiple comparisons test. Graphs appear as mean ± standard deviation (SD). Statistical analysis was performed using the GraphPad Prism 8 software package (GraphPad Software, Inc., La Jolla, CA, USA) and data were considered significantly different if *p* < 0.05.

## Figures and Tables

**Table 1:** P7 body and P14 body and brain weight from sham, hypoxic–ischemic (HI) and URB447-treated (HI+URB447) animals. Data are shown as mean ± standard deviation. * *p* < 0.05; ** *p* < 0.01; *** *p* < 0.001; **** *p* < 0.0001 vs. sham. # *p* < 0.05; ## *p* < 0.01 vs. HI.

Both sexes		**SHAM**	**HI**	**HI+URB447**
	P7 body weight	16.09 ± 0.38	15.51 ± 1.6	15.4 ± 1.20
	P14 body weight	29.45 ± 0.67	23.56 ± 4.25 ****	25.49 ± 3.29 **
	Brain weight	1.12 ± 0.02	0.89 ± 0.08 ****	0.97 ± 0.05 ** #
Males	P7 body weight	16.33 ± 0.31	16.29 ± 1.08	15.36 ± 1.57
	P14 body weight	29.78 ± 0.50	24.86 ± 4.30 *	25.4 ± 3.66
	Brain weight	1.12 ± 0.03	0.92 ± 0.05 ****	0.97 ± 0.05 ***
Females	P7 body weight	15.74 ± 0.03	13.94 ± 1.29 *	15.44 ± 0.73
	P14 body weight	28.95 ± 0.06	22.10 ± 3.92 **	29.5 ± 3.06
	Brain weight	1.11 ± 0.02	0.86 ± 0.08 ****	0.97 ± 0.06 ** ##

## Data Availability

The data presented in this study are available on request from the corresponding author.
